# Annexin 1 Reduces Dermatitis-Induced Itch and Cholestatic Itch through Inhibiting Neuroinflammation and Iron Overload in the Spinal Dorsal Horn of Mice

**DOI:** 10.3390/brainsci14050440

**Published:** 2024-04-28

**Authors:** Tang Li, Lingyue Hu, Chao Qin, Yuanjie Li, Zhenhua Song, Yang Jiao, Chunyan Wang, Wei Cui, Linlin Zhang

**Affiliations:** 1 Department of Anesthesiology and Pain Research Center, The Affiliated Hospital of Jiaxing University, Jiaxing 314001, China; 2 Department of Anesthesiology, Tianjin Medical University General Hospital, Tianjin 300052, China

**Keywords:** Annexin 1, chronic itch, IL-17, iron overload, transferrin receptor 1

## Abstract

The unclear pathogenesis of chronic itch originating from several systemic disorders poses challenges to clinical intervention. Recent studies recapitulate the spinal neurocircuits associated with neuroinflammation and synaptic plasticity responsible for pruriceptive sensations. The resolution of nociception and inflammation by Annexin 1 (ANXA1) has been identified. Given that pain and itch share many neural mechanisms, we employed two mice models of chronic itch to study the underlying targets and therapeutic potential of ANXA1, comprising allergic contact dermatitis-induced itch and cholestatic itch. Herein, we report that spinal expression of ANXA1 is down-regulated in mice with dermatitis-induced itch and cholestatic itch. Repetitive injections of ANXA1-derived peptide Ac2-26 (intrathecal, 10 μg) reduce itch-like scratching behaviors following dermatitis and cholestasis. Single exposure to Ac2-26 (intrathecal, 10 μg) alleviates the established itch phenotypes. Moreover, systemic delivery of Ac2-26 (intravenous, 100 μg) is effective against chronic dermatitis-induced itch and cholestatic itch. Strikingly, Ac2-26 therapy inhibits transferrin receptor 1 over-expression, iron accumulation, cytokine IL-17 release and the production of its receptor IL-17R, as well as astrocyte activation in the dorsal horn of spinal cord in mouse with dermatitis and cholestasis. Pharmacological intervention with iron chelator deferoxamine impairs chronic itch behaviors and spinal iron accumulation after dermatitis and cholestasis. Also, spinal IL-17/IL-17R neutralization attenuates chronic itch. Taken together, this current research indicates that ANXA1 protects against the beginning and maintenance of long-term dermatitis-induced itch and cholestatic itch, which may occur via the spinal suppression of IL-17-mediated neuroinflammation, astrocyte activation and iron overload.

## 1. Introduction

An itch is characterized by an unpleasant somatosensory modality that drives scratching behaviors [1]. An acute itch is usually biological protection against harmful pruritogens and irritants invading the skin, while a chronic itch is an irritating phenotype that has a high prevalence in patients with various systemic diseases, such as dermatological disorders, chronic hepatobiliary diseases, cancer and chronic renal failure [2,3]. Chronic itch constitutes a common and intricate challenge that substantially disrupts emotion, sleep and cognitive function, and exhibits complicated pathological pathways that limit the development of anti-pruriceptive treatments [4,5,6].

Current research has recapitulated the important roles of astrocyte-coding neuroinflammation in the spinal cord neurocircuits for chronic itch pathogenesis, which is associated with excitatory pruriceptive synaptic plasticity and central itch-specific neuronal sensitization [7,8,9]. Specifically, the long-lasting existence of astrocyte activation is uncovered in the dorsal horn of the spinal cord in rodent animals undergoing atopic dermatitis and contact dermatitis [10]. Spinal and pharmacological inhibition of astrocytes is effective against chronic itching behaviors following dry skin damage [11]. Furthermore, proinflammatory cytokine IL-17, as an astrocyte-secreted mediator, is well-known to be implicated in modifications of excitatory sensory synaptic transmission via binding its receptor IL-17RA on neurons in the emergence of opioid-induced hyperalgesia, chronic neuropathic pain, persistent bone cancer pain and acute inflammatory pain [12,13]. Although the up-regulation of keratinocytic IL-17 in dry skin and psoriatic skin has been identified [14], it is still unclear whether the spinal IL-17/IL-17RA pathway is required for chronic itch facilitation. More importantly, neuronal iron overload following neuroinflammation is pivotal a determinant of excitatory synaptic plasticity via dendritic spine morphogenesis in chronic nociceptive states [15,16,17]. However, little has been explored about the interaction between IL-17-dependent neuroinflammation, iron overload and synaptic plasticity in the central circuit mechanisms of chronic itch.

Annexin 1 (ANXA1) is a calcium-regulated phospholipid-binding protein which as gradually been recognized as an endogenous glucocorticoid-regulated anti-inflammatory mediator, which exerts potential therapeutic actions on the resolution of inflammation in several neurological disorders [18,19]. Recently, ANXA1 has gained particular interest due to its emerging properties in pain modulation and resolution [20,21]. In particular, the ANXA1-derived peptide is capable of reducing astrocyte activation and astrogliosis in inflammatory nociception which occurs after acute exposure to a CFA (complete Freund’s adjuvant) [22]. However, the involvement of ANXA1 in chronic itching resolution remains unreported.

We described the therapeutic potential of intrathecal and intravenous ANXA1 mimetic peptide Ac2-26 in chronic itching using two mice models of allergic contact dermatitis-induced itch and cholestatic itch in this pre-clinical research. Spinal alternations of IL-17/IL-17R expression, GFAP level and iron contents were assessed for demonstrating IL-17-dependent astrocyte activation and iron overload as critical contributors to persistent itching and to verify the anti-pruriceptive targets of ANXA1. This research could provide promising perspectives on sensory neurobiology of pathological itching and expedite the development of specific neurotherapies for chronic itch control.

## 2. Materials and Methods

### 2.1. Animals

The Chinese People’s Liberation Army’s Military Medical Science Academy’s Laboratory Animal Center provided the male C57BL/6J mice, which were 8–10 weeks old. Animals were housed in groups of three–five mice per cage at ambient temperature in a cycle of natural day and night with water and food available ad libitum. The experimental procedures and care in the animals were in compliance with the National Institutes of Health Guide for Care and Use of Laboratory Animals. All animal handing protocols were approved by the Institutional Animal Care and Use Committee of Tianjin Medical University General Hospital (IRB2021-DW-72; 25 November 2021; Tianjin, China). Any experiments were conducted following animal acclimation for at least one week.

### 2.2. Reagents

ANXA1-derived mimetic peptide Ac2-26 (Cat. SML3198) and Deferoxamine (DFO; Cat. D9533) were purchased from Sigma-Aldrich (St. Louis, MO, USA). The neutralizing antibody against IL-17 (anti-IL-17, Cat. sc-374218) was from Santa Cruz Biotechnology (Dallas, TX, USA). IL-17R antibody (IL-17R Ab; Cat. MAB4481) was from R&D Systems (Minneapolis, MN, USA). The rationale that choosing dosages of these reagents is according to published literature [13], our preliminary investigations and the manufacturers’ instructions. Before administration, all drugs were stored at −20 °C. For intrathecal injection, a 30-gauge spinal cord puncture was utilized to insert medication (5 µL) into the cerebral spinal fluid between the L4 and L5 levels [23].

### 2.3. ACD-Induced Chronic Itch

An allergic contact dermatitis (ACD) model of chronic itching was established through the application of 1-Fluoro-2,4-dinitrobenzene (DNFB; Cat. D1529; Sigma-Aldrich; St. Louis, MO, USA) onto the neck skin as in previous publications [24,25]. In brief, animals had the nape of their shaved and the surface of their abdomen shaved at least 2 days prior to any experimental sensitization. An acetone and olive oil (4:1) mixture was utilized to dissolve DNFB. Initially, mice were made sensitive by topically applying 50 μL of 0.5% DNFB to their shaved abdomen skin. After 5 days, we challenged the animals by painting the nape of neck skin with 30 μL of 0.25% DNFB, which was then applied on days 2, 4 and 6. The spontaneous scratching activities were observed for 60 min on days 3, 5, 7 and 9 following DNFB application to neck skin. The definition of a scratch was described as raising the hind paw to scratch the applied neck and then lowering it to the ground. For one hour, scratching bouts were counted in a blinded manner.

### 2.4. Cholestasis-Induced Chronic Itch

Using a bile duct ligation (BDL) animal model, created according to the approach outlined [26], surgically led to cholestasis and induced chronic itch. Specifically, using a nose mask, animals were sevoflurane anesthetized (3.0% for induction and 1.5% for operation) on the operating table with the controlled temperature (37 °C). After abdominal shaving and disinfection, 2 cm midline laparotomy was performed, then 4-0 braided silk sutures were utilized to expose and ligate the common bile duct between the right and caudate lobes. Finally, the abdomen was sutured layer by layer after rinsing intraperitoneal cavity with 0.9% sodium chloride injection. Sham operation was performed identically with no BDL. Cholestasis was verified by the intact ligature, increased serum level of total bilirubin as well as proximal dilation of the common bile duct on day 14 after BDL surgery. Scratching behaviors were recorded for one hour and quantified as previous reports in a blinded manner.

### 2.5. Western Blot

The animals were deeply anesthetized with sevoflurane (3%). The dorsal horns of the cervical (for ACD model) and lumbar (for BDL model) segments in the spinal cord were quickly removed and cryopreserved in liquid nitrogen. The sample was homogenized mechanically in ice-cold RIPA buffer that contained PMSF (Abcam, Cambridge, UK). The bicinchoninic acid test method was used to assess the amount of protein present. Using a membrane coated with a monoclonal mouse anti-β-actin antibody (1:5000; Sigma-Aldrich), the loading and blotting of an identical quantity of total proteins were confirmed. Following resolution on a 10% SDS-PAGE gel, the samples were transferred to nitrocellulose membranes, and probed with rabbit antibodies against ANXA1 (1:2000, ab137745, Abcam), transferrin receptor 1 (TfR1, 1:2000, ab214039, Abcam), iron regulatory protein 1 (IRP1, 1:2000, ab236773, Abcam) and divalent metal transporter 1 (DMT1, 1:2000, ab262715, Abcam). After which secondary antibodies coupled with horseradish peroxidase (1:2000, Jackson Immuno Research, West Grove, PA, USA) were incubated. Enhanced chemiluminescence (Thermo Scientific, Rockford, IL, USA) was used to visualize the membrane-bound secondary antibodies, and Media Cybernetics Inc.’s Image-Pro Plus software (Version 6.0) was used to quantify the results.

### 2.6. ELISA Analysis

An enzyme-linked immunosorbent assay (ELISA) was utilized to measure the concentrations of IL-17 (ab100702, Abcam) and GFAP (ab233621, Abcam) in the cervical and lumbar segments of the spinal cords. Protease and phosphatase inhibitors were added to a lysis buffer that was used to homogenize spinal cord tissues. After centrifuging tissue samples for 10 min at 12,500× *g*, the supernatant was gathered. To ascertain protein concentrations, we used the BCA Protein Assay (Pierce, Rockford, IL, USA). A 96-well plate containing 100 μg of the sample proteins was used for each experiment. Every ELISA experiment complied with the manufacturer’s instructions. Using an ELISA plate reader (Bio-Rad, Hercules, CA, USA) set to operate at the wavelength of 450 nm, the optical densities of the samples were determined. The levels of GFAP and IL-17 were then computed using standard curves and normalized to the total protein levels.

### 2.7. Real-Time qPCR

The levels of IL-17R mRNA in the dorsal horns of the spinal cord’s cervical and lumbar segments were identified. Total mRNA extraction was carried out in accordance with manufacturer’s instructions, and the RNA 4 Aqueous kit (Ambion Inc., Austin, TX, USA) was used for purification. Following the assessment of concentration, each mRNA sample underwent a reverse transcriptase reaction using a Retroscript kit from Ambion Inc. (Austin, TX, USA). The template for creating first-strand cDNA was total mRNA. The API Prism 7900HT Sequence Detection system was used to perform RT-qPCR with three independent repetitions in accordance with the SYBER Green PCR Master Mix (Applied Biosystems, Foster City, CA, USA) instructions. The reaction protocols consisted of 2 min at 50 °C, 10 min at 95 °C, 40 cycles of 15 s at 95 °C and 60 s at 60 °C. The normalized curve was used to calculate each gene’s expression. According to the delta-delta-Ct method, quantitative standardization was carried out in each sample using the expression of GAPDH (glyceraldehydes 3-phosphate dehydrogenase) as an endogenous control [27]. The data were shown as fold change relative to the control. The following forward and reverse primers were used: IL-17R (Forward: 5′-TACCACAGTTCCCAAGCC AGTT-3′; Reverse: 5′-GGGGAGTCAGGTCTGCTACG-3′), and GAPDH mRNA (5′-AGGTCGGTGTGAACGGATTTG-3′; 5′-TGTA GACCATGTAGTTGAGGTCA-3′).

### 2.8. Iron Content Assay

By digesting the tissues, the iron content of the spinal dorsal horns was measured using a flame atomic absorption spectrophotometer (Shimadzu AA-6800, Kyoto, Japan) at 248.3 nm [17,27]. Samples (0.1–0.2 g) were dried at 60 °C for 12 h, then digested with 1 mL of 60% nitric acid at 100 °C in a water bath for 2 h. After adding 0.5 mL of hydrogen peroxide, the process was continued for an additional 0.5 h at boiling point. This process was performed to obtain dry mass. Prior to computation, the completely dissolved residues were diluted to 10 mL using double-distilled water. By comparing the absorbance to a variety of standard values of FeSO_4_, atomic iron levels were determined.

### 2.9. Statistical Analysis

We utilized SPSS 21.0 software (SPSS, Inc., Chicago, IL, USA) for all of the statistical analyses. The findings were displayed using box-and-whiskers plots, where the 5th and 95th percentiles were displayed in the “whisker” and the median, 25th, and 75th quartiles were shown in the “box”. The box-and-whiskers plots were overlayed with individual data points. All behavioral and biochemical data were analyzed using the one-way or two-way ANOVA followed by Bonferroni post hoc comparisons, as well as the two-tailed Student’s *t* test. The threshold for statistical significance was *p* < 0.05.

## 3. Results

### 3.1. Intrathecal Administration of ANXA1-Derived Peptide Ac2-26 Reduces the Onset and Persistence of ACD-Induced Long-Term Itching

**Figure 1:**
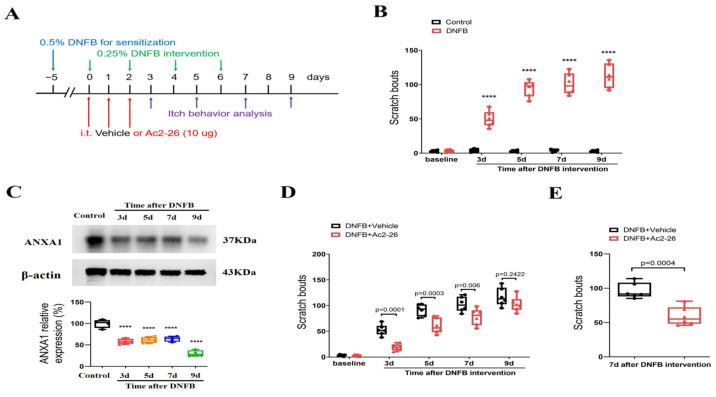
Spinal therapy of ANXA1-derived peptide Ac2-26 reduces dermatitis-induced chronic itch in mice. (**A**) Experimental design for intrathecal (i.t.) treatment with Ac2-26 in DNFB-induced chronic pruritus. (**B**) Scratching bouts after DNFB exposure. *n* = 6 mice/group. (**C**) The expression of ANXA1 in the spinal dorsal horn after DNFB exposure was measured. *n* = 4 mice/group. (**D**) Pre-administrations of Ac2-26 prevent DNFB-induced scratching behaviors. *n* = 6 mice/group. (**E**) Single injection of Ac2-26 (i.t., 10 μg) on day 7 after DNFB exposure attenuates the established persistent scratching behaviors. *n* = 6 mice/group. All behavioral and biochemical results are expressed as medians with interquartile ranges and individual data plots. **** *p* < 0.0001 vs. group Control.

First, animals were challenged with DNFB to induce long-lasting ACD (Figure 1A). Herein, we found that the scratching behavior was unaltered in the olive oil-treated control mice when compared with baseline, suggesting that dissolvent did not cause itch (Figure 1B). However, DNFB exposure elicited a rapid (<3 days) and persistent (>9 days, the last observation day) increase in scratching behaviors (F (1, 50) = 848.1, *p* < 0.0001, *n* = 6, two-way ANOVA, Figure 1B), suggesting that a chronic itch was successfully induced and sustained following ACD. Furthermore, spinal expression of ANXA1 was dramatically decreased from 3 days to 9 days following DNFB application (F (4, 15) = 46.02, *p* < 0.0001, *n* = 4, one-way ANOVA, Figure 1C), which resembled the pattern of behaviors related to persistent itching.

Then, to test the role of ANXA1 in chronic pruritus, the animals were given three shots of Ac2-26 (intrathecal, 10 μg) daily for three consecutive days from day 0 to day 2 (in the initial stage) following DNFB treatment (Figure 1A). Interestingly, repetitive injections of Ac2-26 reduced DNFB-induced scratching behaviors (F (1, 50) = 42.4, *p* < 0.0001, *n* = 6, two-way ANOVA, Figure 1D), and its anti-pruriceptive effects strongly lasted for a minimum of five days following three shots. Furthermore, we looked at the effectiveness of post-treatment with Ac2-26 at existing pruritus. As expected, a single delivery of Ac2-26 (intrathecal, 10 μg) on day 7 (in the late phase) after DNFB application exhibited a considerable alleviation of the established itch in mice with ACD (*p* = 0.0004, two-tailed Student’s *t* test, Figure 1E). Taken together, the results show that intrathecal Ac2-26 therapy confers protection against ACD-induced persistent itch behaviors.

### 3.2. ANXA1-Derived Peptide Ac2-26 Inhibits Spinal IL-17 and IL-17R over-Expression, Astrocyte Activation and Iron Overload in ACD Mice

**Figure 2:**
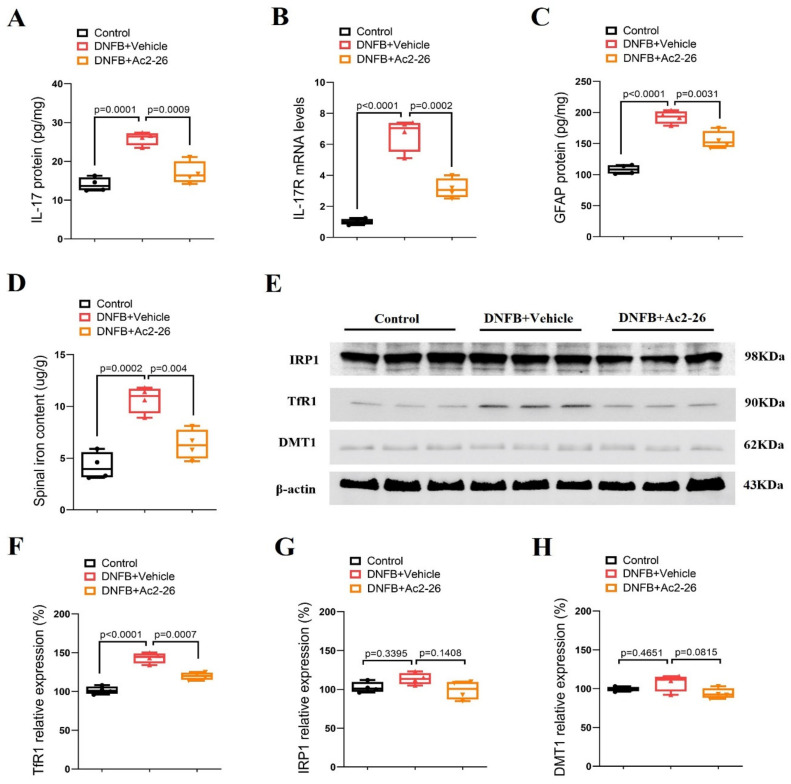
ANXA1-derived peptide Ac2-26 down-regulates spinal IL-17 release, IL-17R expression, astrocyte activation and iron overload after dermatitis in mice. Repetitive injections of Ac2-26 (intrathecal, 10 μg) daily for three consecutive days were performed from day 0 to day 2 following DNFB exposure. The dorsal horns of spinal cord were collected on day 3 following DNFB exposure. (**A**) The expression of IL-17 in the spinal dorsal horn was measured by ELISA assay. (**B**) The expression of IL-17R in the spinal dorsal horn was measured by RT-qPCR. (**C**) The expression of GFAP in the spinal dorsal horn was measured by ELISA assay. (**D**) The iron concentration in the spinal dorsal horn was measured. (**E**–**H**) Western blot showed the changes of spinal iron metabolism-related proteins (TfR1, IRP1 and DMT1) after DNFB exposure and Ac2-26 treatment, respectively. *n* = 4 mice/group. All biochemical results are expressed as medians with interquartile ranges and individual data plots.

Astrocytes that are involved in neuroinflammatory process and dorsal horn synaptic plasticity in the spinal cord are one of the most important contributors to the pathogenesis of persistent pruritus [7,8,9]. Biochemical experiments were employed to quantitatively measure spinal IL-17, IL-17R, GFAP, iron content and iron metabolism-related proteins (TfR1, IRP1 and DMT1). It was detected that the spinal expressions of IL-17, IL-17R and GFAP were dramatically elevated on day 3 after DNFB intervention (*p* < 0.05, *n* = 4, Figure 2A–C). In parallel, we observed that DNFB caused a robust increase in the iron concentration and an overexpression of TfR1 (*p* < 0.05, *n* = 4, Figure 2D–F) while the IRP1 and DMT1 levels were not changed (Figure 2E,G,H). Intriguingly, pre-treatment with Ac2-26 (intrathecal, 10 μg) suppressed the up-regulation of IL-17, IL-17R and GFAP expressions following DNFB exposure (*p* < 0.05, *n* = 4, Figure 2A–C). Also, TfR1-mediated iron overload was impaired by pre-administration of Ac2-26 (*p* < 0.05, *n* = 4, Figure 2D–F). Collectively, these specific findings imply that the inhibitory effects of ANXA1 on ACD-induced chronic itch might be associated with reducing IL-17-mediated astrocyte activation and TfR1-caused iron accumulation in the dorsal horn of the spinal cord.

### 3.3. Intrathecal Administration of ANXA1-Derived Peptide Ac2-26 Prevents and Ameliorates Chronic Cholestatic Itch

**Figure 3:**
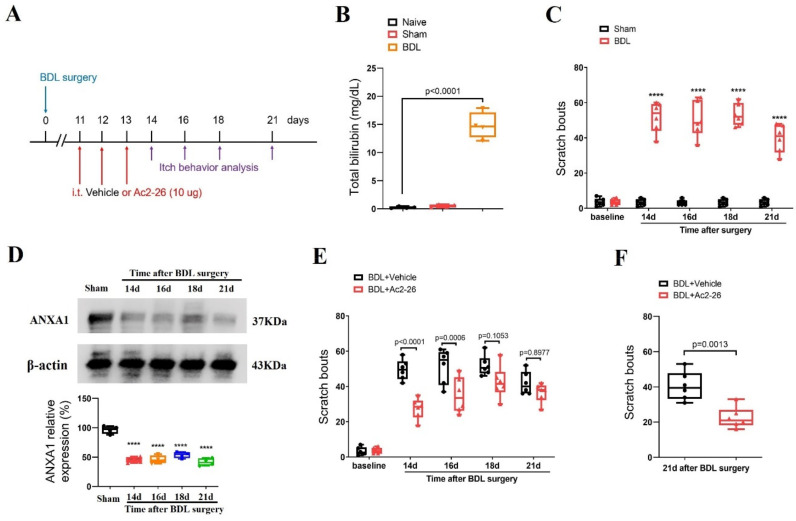
Spinal therapy with ANXA1-derived peptide Ac2-26 reduces cholestatic itch in mice. (**A**) Experimental design for intrathecal (i.t.) treatment with Ac2-26 in BDL-induced chronic pruritus. (**B**) Serum levels of total bilirubin were measured after sham and BDL surgery. *n* = 4 mice/group. (**C**) Scratching bouts after BDL surgery. *n* = 6 mice/group. (**D**) The expression of ANXA1 in the spinal dorsal horn after BDL surgery was measured. *n* = 4 mice/group. (**E**) Pre-administration of Ac2-26 prevents BDL-induced scratching behaviors. *n* = 6 mice/group. (**F**) Single injection of Ac2-26 (i.t., 10 μg) on day 21 after BDL surgery attenuates the established persistent scratching behaviors. *n* = 6 mice/group. All behavioral and biochemical results are expressed as medians with interquartile ranges and individual data plots. **** *p* < 0.0001 vs. sham group.

Next, to evaluate the substantial contribution of ANXA1 to cholestatic itching, repetitive injections of Ac2-26 (intrathecal, 10 μg) were performed every day between days 11 and 13 (in the initial stage) after BDL surgeries (Figure 3A). Herein, BDL operation increased serum level of total bilirubin when compared with sham surgery (F (2, 9) = 145.3, *p* < 0.0001, *n* = 4, one-way ANOVA, Figure 3B), suggesting the establishment of the cholestasis model. Compared to the sham mice, the BDL mice exhibited a persistent increase in spontaneous scratching behaviors from days 14 to day 21 (the last examination day) after surgeries, suggesting the generation and development of a chronic cholestatic itch (F (1, 50) = 656.9, *p* < 0.0001, *n* = 6, two-way ANOVA, Figure 3C). Strikingly, Western blot analysis showed a dramatic and long-lasting decrease in ANXA1 expression in the spinal dorsal horn (F (4, 15) = 57.39, *p* < 0.0001, *n* = 4, one-way ANOVA, Figure 3D), which is associated with the time course of persistent cholestatic itching.

Moreover, pre-administrations of Ac2-26 reduced BDL-induced scratching behaviors (F (1, 50) = 36.14, *p* < 0.0001, *n* = 6, two-way ANOVA, Figure 3E), and its anti-pruriceptive effects strongly lasted for at least 3 days after three injections. In parallel, a single injection of Ac2-26 (intrathecal, 10 μg) on day 21 (in the late phase) after the BDL surgeries also attenuated the existing itch-like scratching behaviors in mice with cholestasis (*p* = 0.0013, two-tailed Student’s *t* test, Figure 3F). Thus, intrathecal Ac2-26 therapy confers protection against BDL-induced persistent cholestatic itching.

### 3.4. ANXA1-Derived Peptide Ac2-26 Inhibits Spinal IL-17 and IL-17RA over-Expression, Astrocyte Activation and Iron Overload in Cholestasis Mice

**Figure 4:**
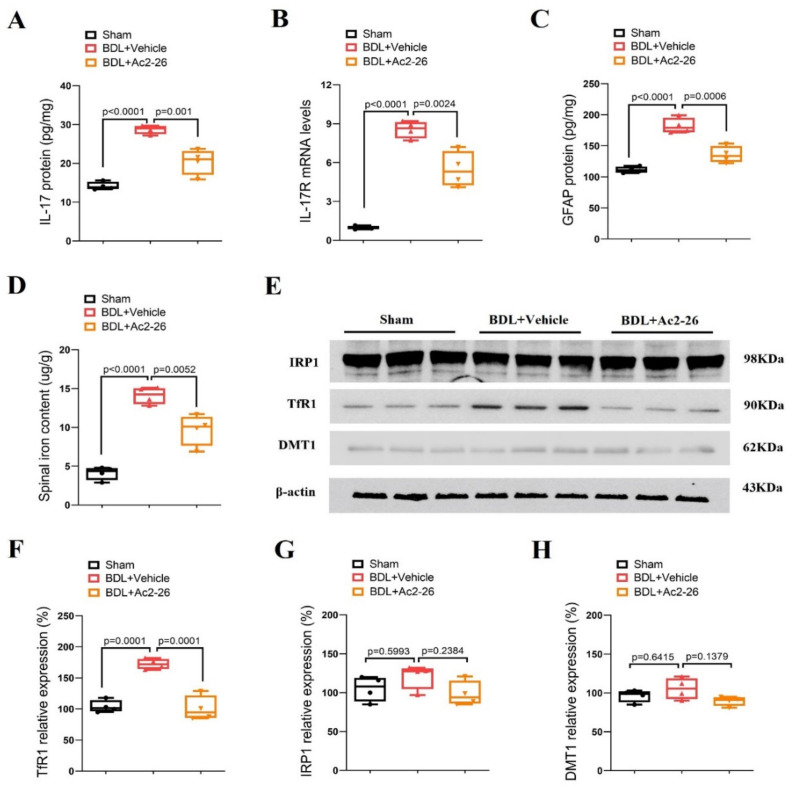
ANXA1-derived peptide Ac2-26 down-regulates spinal IL-17 release, IL-17R expression, astrocyte activation and iron overload after cholestasis in mice. Repetitive injections of Ac2-26 (intrathecal, 10 μg) daily for three consecutive days were performed from day 11 to day 13 following BDL surgery. The dorsal horns of spinal cords were collected on day 14 following BDL surgery. (**A**) The expression of IL-17 in the spinal dorsal horn was measured by ELISA assay. (**B**) The expression of IL-17R in the spinal dorsal horn was measured by RT-qPCR. (**C**) The expression of GFAP in the spinal dorsal horn was measured by ELISA assay. (**D**) The iron concentration in the spinal dorsal horn was measured. (**E**–**H**) Western blot showed the changes in spinal iron metabolism-related proteins (TfR1, IRP1 and DMT1) after BDL surgery and Ac2-26 treatment, respectively. *n* = 4 mice/group. All biochemical results are expressed as medians with interquartile ranges and individual data plots.

Spinal levels of IL-17, IL-17R, GFAP, iron metabolism-related proteins (TfR1, IRP1 and DMT1) and iron content were also evaluated to verify if they are involved in the anti-pruritus effects of ANXA1 on cholestatic itch. Interestingly, the robust elevation of IL-17, IL-17R and GFAP expression was observed in the spinal dorsal horns of the animals on 14 days after BDL surgeries (*p* < 0.05, *n* = 4, Figure 4A–C). Strikingly, pre-administrations of Ac2-26 (intrathecal, 10 μg) blocked the up-modulation of spinal IL-17, IL-17R and GFAP expressions following BDL surgeries (*p* < 0.05, *n* = 4, Figure 4A–C). Furthermore, Ac2-26 reduced iron overload and TfR1 overexpression in cholestasis mice (*p* < 0.05, *n* = 4, Figure 4D–F), but no changes in IRP1 and DMT1 levels was detected among the groups (Figure 4E,G,H). All of these biochemical results imply that ANXA1 might control chronic cholestatic itching via spinal inhibition of IL-17-mediated astrocyte activation and TfR1-dependent iron overload.

### 3.5. Spinal Neutralization of IL-17/IL-17R Alleviates Dermatitis-Induced Itching and Cholestatic Itching via Reducing TfR1-Dependent Iron Overload

**Figure 5:**
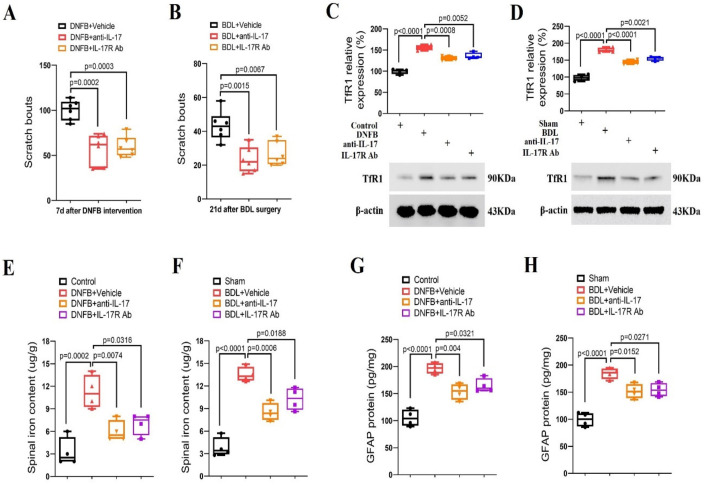
Spinal inhibition of IL-17 cascade reduces dermatitis-induced chronic itch and cholestatic itch in mice. (**A**) Intrathecal injection of anti-IL-17 (2 μg) and IL-17R Ab (2 μg) were performed on day 7 following DNFB intervention. The therapy with anti-IL-17 and IL-17R Ab attenuated the established persistent scratching behaviors. *n* = 6 mice/group. (**B**) Intrathecal injections of anti-IL-17 (2 μg) and IL-17R Ab (2 μg) were performed on day 21 following BDL operation. The therapy with anti-IL-17 and IL-17R Ab attenuated the established persistent scratching behaviors. *n* = 6 mice/group. (**C**–**F**) Spinal expression of TfR1 and iron concentration after anti-IL-17 and IL-17R Ab treatment were measured in mice with DNFB exposure and BDL surgery, respectively. *n* = 4 mice/group. (**G**,**H**) The expression of GFAP in the spinal dorsal horn was measured by ELISA assay. *n* = 4 mice/group. All behavioral and biochemical results are expressed as medians with interquartile ranges and individual data plots.

To further evaluate if spinal IL-17/IL-17R cascades are required for the pathogenesis of long-term pruriceptive behaviors, anti-IL-17 (intrathecal, 2 μg) and IL-17R Ab (intrathecal, 2 μg) were injected once on day 7 following DNFB intervention. Strikingly, both anti-IL-17 and IL-17R Ab induced a significant reduction in spontaneous scratching activities (F (2, 15) = 19.53, *p* < 0.0001; *n* = 6, one-way ANOVA, Figure 5A) in the DNFB-treated animals. Meanwhile, single injection of anti-IL-17 (intrathecal, 2 μg) and IL-17R Ab (intrathecal, 2 μg) on day 21 following BDL surgeries attenuated the current scratching activities (F (2, 15) = 11.18, *p* = 0.0011; *n* = 6, one-way ANOVA, Figure 5B). More importantly, anti-IL-17 and IL-17R Ab decreased spinal TfR1 over-expression and iron accumulation in animals undergoing DNFB application and BDL surgery (all *p* < 0.05, *n* = 4, Figure 5C–F). Also, pharmacological blockage of the IL-17 pathway inhibited spinal astrocyte activation in chronic itch mice (*p* < 0.05, *n* = 4, Figure 5G,H). Collectively, these results demonstrate the implication of spinal IL-17/IL-17R signaling in the development of dermatitis-induced chronic itch and cholestatic itch via modulating astrocyte activation and iron overload.

### 3.6. Iron Chelation Suppresses Dermatitis-Induced Chronic Itch and Cholestatic Itch

**Figure 6:**
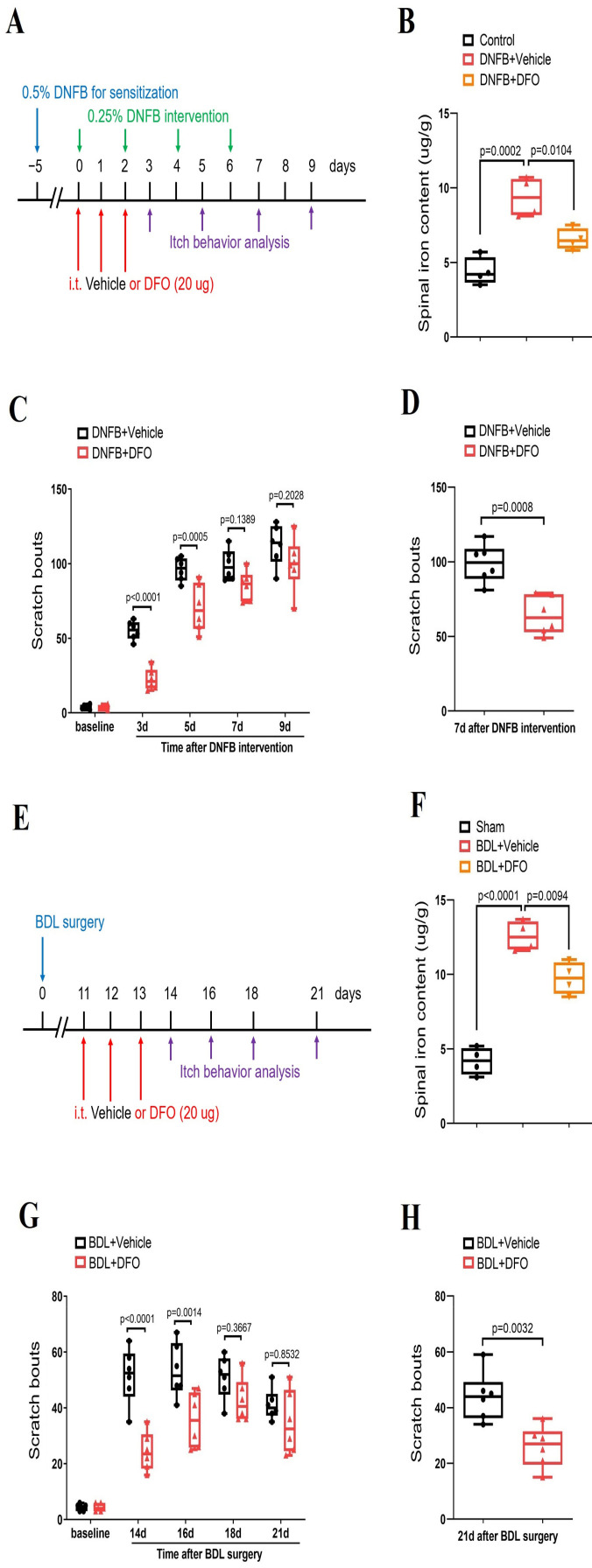
Iron chelation reduces dermatitis-induced chronic itch and cholestatic itch in mice. (**A**) Experimental design for intrathecal (i.t.) treatment with iron chelator DFO in DNFB-induced chronic pruritus. (**B**) Spinal iron concentration after DNFB and DFO treatment was measured. The dorsal horns of spinal cord were collected on day 3 following DNFB exposure. *n* = 4 mice/group. (**C**) Pre-administration of DFO prevented DNFB-induced scratching behaviors. *n* = 6 mice/group. (**D**) Single injection of DFO (i.t., 20 μg) on day 7 after DNFB exposure attenuates the established persistent scratching behaviors. *n* = 6 mice/group. (**E**) Experimental design for intrathecal (i.t.) treatment with iron chelator DFO in BDL-induced chronic pruritus. (**F**) Spinal iron concentration after BDL surgery and DFO treatment was measured. The dorsal horns of spinal cord were collected on day 14 following BDL surgery. *n* = 4 mice/group. (**G**) Pre-administration of DFO prevented BDL-induced scratching behaviors. *n* = 6 mice/group. (**H**) Single injection of DFO (i.t., 20 μg) on day 21 after BDL surgery attenuates the established persistent scratching behaviors. *n* = 6 mice/group. All behavioral and biochemical results are expressed as medians with interquartile ranges and individual data plots.

Next, we sought to test whether iron accumulation is a critical step for the modulation of neuronal activity and central pruriceptive sensitization in itch conditions. To tackle this, iron chelator DFO (intrathecal, 20 μg) was administered (Figure 6A–H). Herein, we found that repetitive DFO therapy effectively inhibited DNFB-induced spinal iron accumulation (F (2, 9) = 24.05, *p* = 0.0002, *n* = 4, one-way ANOVA, Figure 6A,B) and prevented the initiation of DNFB-induced itch-like scratching behaviors (F (1, 50) = 39.25, *p* < 0.0001, *n* = 6, two-way ANOVA, Figure 6A,C). Simultaneously, repeated administrations of DFO reduced BDL-induced spinal iron accumulation (F (2, 9) = 73.04, *p* < 0.0001, *n* = 4, one-way ANOVA, Figure 6E,F) and spontaneous scratching behaviors (F (1, 50) = 34.31, *p* < 0.0001, *n* = 6, two-way ANOVA, Figure 6E,G). Surprisingly, a single injection of DFO was able to attenuate the established itch in the DNFB model (*p* = 0.0008, *n* = 6, two-tailed Student’s *t* test, Figure 6D) and BDL model (*p* = 0.0032, *n* = 6, two-tailed Student’s *t* test, Figure 6H). Thus, these results demonstrate that iron overload in the spinal cord itch neurocircuit is required for chronic pruritus development. 

### 3.7. Systemic Therapy of ANXA1-Derived Peptide Ac2-26 Controls Dermatitis-Induced Chronic Itch and Cholestatic Itch

**Figure 7:**
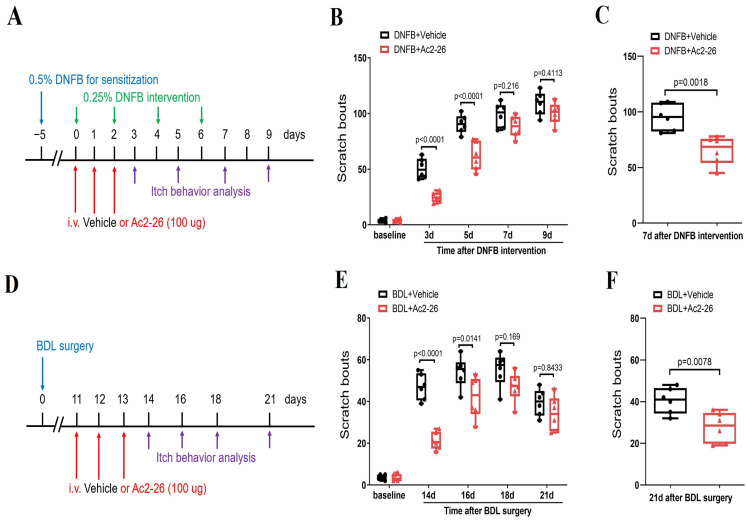
Systemic administration of ANXA1-derived peptide Ac2-26 reduces dermatitis-induced chronic itch and cholestatic itch in mice. (**A**) Experimental design for intravenous (i.v.) treatment with Ac2-26 in DNFB-induced chronic pruritus. (**B**) Pre-administration of Ac2-26 prevented DNFB-induced scratching behaviors. *n* = 6 mice/group. (**C**) Single injection of Ac2-26 (i.v., 100 μg) on day 7 after DNFB exposure attenuates the established persistent scratching behaviors. *n* = 6 mice/group. (**D**) Experimental design for intravenous (i.v.) treatment with Ac2-26 in BDL-induced chronic pruritus. (**E**) Pre-administration of Ac2-26 prevented BDL-induced scratching behaviors. *n* = 6 mice/group. (**F**) Single injection of Ac2-26 (i.v., 100 μg) on day 21 after BDL surgery attenuates the established persistent scratching behaviors. *n* = 6 mice/group. All behavioral results are expressed as medians with interquartile ranges and individual data plots.

Considering the low translational value of Ac2-26 in clinical patients because of its delivery methods (intrathecal injection) to mice, we finally explored whether systemic Ac2-26 (intravenous, 100 μg) treatment is effective against chronic pruritus syndrome (Figure 7A–F). Interestingly, intravenous pre-administrations of Ac2-26 prevented DNFB-induced persistent scratching behaviors (F (1, 50) = 43.74, *p* < 0.0001, *n* = 6, two-way ANOVA, Figure 7A,B) and BDL-induced scratching phenotypes (F (1, 50) = 36.62, *p* < 0.0001, *n* = 6, two-way ANOVA, Figure 7D,E). Meanwhile, a single injection of Ac2-26 following the intravenous route ameliorated the existing DNFB-induced itch-like activities (*p* = 0.0018, two-tailed Student’s *t* test, Figure 7C) and BDL-induced spontaneous scratching bouts (*p* = 0.0078, two-tailed Student’s *t* test, Figure 7F)

## 4. Discussion

A chronic itch, as an uncomfortable symptom of multiple pathological states, results from peripheral and central sensitization of the pruriceptive neurocircuitry in the peripheral nerve, primary sensory neurons, spinal dorsal horn and brain [28,29,30]. Given that spinal neuroinflammation caused by synaptic alternations is considered a pivotal steps in chronic pruriceptive perception [7,9,31], there is great interest in how neuroinflammation in the spinal dorsal horn is initiated and how it is resolved.

Astrocytes are recognized as having well-established roles in the neuroinflammatory responses to neuropsychological diseases and neuropathic disorders [32,33]. Of these, astrogliosis and astrocyte activation in the dorsal horn is responsible for the long-lasting mechanical allodynia behaviors following spared nerve damage [34]. A pharmacological reduction in the astrocyte activation in the dorsal horn is capable of alleviating chemotherapy-caused neuropathic pain through the resolution of neuroinflammation [35]. However, the specific molecular mechanism of astrocyte activation has yet to be investigated. Recently, it has been found that spinal IL-17 overexpression facilitates astrocyte activation, induces neuronal hyperexcitability and elicits mechanical allodynia in naïve mice [36]. Also, the attenuation of both peripheral neuropathic nociception and bone cancer nociception after central neutralization of spinal IL-17 was demonstrated to occur through the blocking of astrocyte activation and astrogliosis [13]. Given the important role of astrocytes in persistent pruriceptive syndromes [7,9], we examined if spinal IL-17 contributes to chronic itch behaviors in our animal models. Herein, we firstly found the spinal elevation of IL-17 production as well as the GFAP level in mice with an ACD-induced persistent itch and a chronic cholestatic itch. Strikingly, this is the first study to report that the pharmacological neutralization of IL-17 via intrathecal injection reduces dermatitis-induced spontaneous itch behaviors and cholestatic itch through inhibiting astrocyte activation. However, how IL-17 cascades in the dorsal horn are activated and sustained in the pathophysiology of long-term itching needs to be explained. Anyway, these results strongly suggest the requirement of spinal IL-17 signaling for persistent pruritus states following dermatitis and cholestasis, pointing to the possibility that abating spinal astrocyte-involved neuroinflammation might provide novel neurotherapeutics for chronic pruritus syndromes.

Many reports have discussed the contribution of ANXA1 to the resolution of neuroinflammation and neuroprotection. Specifically, ANXA1 has been found to suppress cerebral inflammation to alleviate cerebral ischemia-reperfusion injury in rodent models [37,38]. Furthermore, ANXA1 reduced memory deficits by resolving inflammation in the hippocampus of mice undergoing perioperative neurocognitive disorders [39]. Glial cells-mediated neuroinflammation, brain edema and neurological function were attenuated by ANXA1 in mice following intracerebral hemorrhage [19]. Of note, recent studies have disclosed the inhibition of astrocyte activation and the subsequent reduction in pro-inflammatory mediators due to ANXA1 in animal models of streptococcus suis-induced meningitis [40], pilocarpine-induced status epilepticus [41] and complete Freund’s adjuvant-induced thermal nociception and remifentanil-induced hyperalgesia [21,22]. Yet, the targets and therapeutic potential of ANXA1 in persistent itch are still far from clear. Ac2-26 is the pharmacophore N-terminal peptide of ANXA1 and is widely believed to mimic multiple bioactivities of ANXA1, which gradually recognizes Ac2-26 as an appropriate candidate for research into rodent animals’ responses to ANXA1 [37,41]. To the best of our understanding, this paper shows, for the first time, that both dermatitis and cholestasis cause a persistent decrease in ANXA1 expression, which is consistent with the scratching behaviors associated with a chronic itch. Furthermore, this is the first research in which both spinal (intrathecal) and systemic (intravenous) therapies with ANXA1-derived peptide Ac2-26 have been found to prevent and attenuate persistent itch-like scratching behaviors following ACD and cholestasis. Additionally, ANXA1 suppressed IL-17 production, IL-17R expression and astrocyte activation in the spinal dorsal horns of mice with dermatitis and cholestasis. Overall, these behavioral and biochemical data emphasize that ANXA1 restricts the initiation and maintenance of long-term pruritus via blocking spinal IL-17-mediated astrocyte activation and subsequently resolving neuroinflammation in both dermatitis and cholestasis. Nevertheless, how ANXA1 down-modulates spinal IL-17/IL-17R cascades requires further investigation. In addition, astrocyte activation is a critical promoter of plasticity in excitatory sensory synapses through the crosstalk of IL-17 (in astrocytes) and its receptor IL-17R (in neurons) [36]. Thus, it is of great interest to identify the intracellular events and downstream signaling pathways in neurons in chronic itch settings.

Dysregulation of iron homeostasis in neurons causes neurotoxicity and neuroplasticity, which is a key factor in the neurobiology of disorders [42,43]. More strikingly, neuronal iron overload underlies neuroinflammation-induced synaptic and behavioral plasticity [15,17]. A complete complement of iron proteins mediates intracellular iron homeostasis in the central nervous system, comprising IRP1, DMT1 and TfR1. Neurons uptake iron through TfR1 and DMT1 while IRP1 can regulate the translation of iron proteins [44]. Our previous study has highlighted the requirement of DMT1-dependent neuronal iron accumulation for spine generation and excitatory neuroplasticity in the pathogenesis of tibial fractures-associated long-term pain and remifentanil-caused hypernociception [27,45,46]. TfR1-mediated iron overload is also identified to be one crucial target of astrocyte-dependent neuroinflammation in mechanical allodynia following orthopedic surgeries [17]. However, the overall link between ANXA1, IL-17, astrocyte activation and iron overload in chronic pruritus is unknown. This present research is the first to find out that spinal upregulations of TfR1 expression and iron content after dermatitis and cholestasis are reversed by ANXA1, IL-17 neutralization and IL-17R antagonism, respectively. Additionally, this study offers the first proof that the impairment of spinal iron overload by iron chelator therapy attenuates ACD-induced chronic itch and persistent cholestatic itch. Together, this detailed evidence suggest that spinal IL-17 and IL-17R signaling causes astrocyte activation and TfR1-dependent neuronal iron overload in the development of a chronic itch after dermatitis or cholestasis. While our findings have provided a fundamental insight into the complex neuropathogenesis of chronic itching, further mechanistic research is required to discover the specific connections between these molecular cascades, neural networks and behaviors in pruritus sensation within neuromodulation.

There are several limitations to be considered in our research. One is that no female animals were used to test whether the protective effects of ANXA1 are sex-dependent, which should be taken into consideration in the future. Another limitation is that we failed to investigate whether ANXA1 can treat allergic contact dermatitis and cholestasis. Furthermore, we only tested the effects of ANXA1 on the synaptic plasticity and neuroinflammation in the spinal dorsal horn following chronic itch, but the changes in the peripheral nerve terminals, dorsal root ganglion and brain require further investigation. Also, we did not evaluate whether ANXA1 is effective against acute and chronic itching with other etiologies, such as opioid-induced acute itching, cancer-induced chronic itching and renal failure-induced chronic itching, which is important for translating these findings to clinical patients.

## 5. Conclusions

The critical findings of this current research are the following: First, intrathecal pre-treatment with Ac2-26 prevents persistent scratching behaviors after ACD and cholestasis. Second, intrathecal post-treatment with Ac2-26 ameliorates the existing dermatitis-induced persistent itch and cholestatic itch. Third, Ac2-26 intervention abates IL-17 release, IL-17R expression, astrocyte activation and TfR1-dependent iron overload in the spinal dorsal horns of mice with ACD and cholestasis. Fourth, spinal IL-17 and IL-17R neutralization are protective against dermatitis-induced itch and cholestatic itch through diminishing TfR1-dependent iron overload. Fifth, pharmacological iron chelation restrains the initiation and persistence of pruritus in ACD and cholestasis mice. Sixth, systemic treatment with Ac2-26 confers effective protection against persistent itch states in mice with ACD and cholestasis. Collectively, the aforementioned work unveils a previously unidentified contribution of ANXA1 on preventing and relieving dermatitis-induced chronic itching and cholestatic itching through the inhibitory modulation of IL-17/IL-17R expression, astrocyte activation and TfR1-dependent iron overload in the spinal dorsal horn in mice, which has important implications for neuroinflammation control and itch management in clinical settings.

## Data Availability

All data relevant to the study are included in the article for figures. Data are available from the corresponding author upon reasonable request.
